# Borderline pulmonary arterial pressure in systemic sclerosis patients: a post-hoc analysis of the DETECT study

**DOI:** 10.1186/s13075-014-0493-1

**Published:** 2014-12-10

**Authors:** Scott H Visovatti, Oliver Distler, J Gerry Coghlan, Christopher P Denton, Ekkehard Grünig, Diana Bonderman, Ulf Müller-Ladner, Janet E Pope, Madelon C Vonk, James R Seibold, Juan-Vicente Torres-Martin, Martin Doelberg, Harbajan Chadha-Boreham, Daniel M Rosenberg, Vallerie V McLaughlin, Dinesh Khanna

**Affiliations:** Division of Cardiology, Department of Medicine, University of Michigan, Ann Arbor, MI USA; Division of Rheumatology, University Hospital Zurich, Zurich, Switzerland; Cardiology Department, Royal Free Hospital, London, UK; Centre for Rheumatology, Royal Free Hospital, London, UK; Centre for Pulmonary Hypertension, University Hospital, Heidelberg, Germany; Department of Internal Medicine II, Division of Cardiology, Medical University of Vienna, Vienna, Austria; Department of Rheumatology and Clinical Immunology, Justus-Liebig University, Giessen, Germany; Department of Medicine, Division of Rheumatology, Western University of Canada, London, ON Canada; Department of Rheumatology, Radboud University Medical Centre, Nijmegen, The Netherlands; Scleroderma Research Consultants LLC, Litchfield, CT USA; Actelion Pharmaceuticals Ltd, Allschwil, Switzerland; Division of Rheumatology, Department of Internal Medicine, University of Michigan, Ann Arbor, Michigan USA; Division of Rheumatology, Department of Internal Medicine, University of Michigan Scleroderma Program, 300 North Ingalls Street, Suite 7C27, Ann Arbor, MI 48109 USA

## Abstract

**Introduction:**

Patients with mean pulmonary artery pressures (mPAP) of 21 to 24 mm Hg have a so-called borderline elevation of mPAP (BoPAP)—a condition thought to represent early-stage pulmonary arterial vasculopathy. Based on the DETECT study, this post-hoc analysis examined patient characteristics of systemic sclerosis (SSc) patients with normal mPAP, BoPAP and elevated mPAP, fulfilling pulmonary arterial hypertension (PAH) criteria.

**Methods:**

Adult patients with a duration of SSc more than 3 years, a diffusing capacity of the lung for carbon monoxide less than 60% predicted, and no previous diagnosis of any form of pulmonary hypertension (PH) underwent screening tests followed by right heart catheterization. Subjects were divided into three groups: normal mPAP, BoPAP, and PAH. Exploratory comparative and binary logistic regression analyses were performed for the BoPAP versus normal mPAP and PAH versus BoPAP groups.

**Results:**

Of 244 patients evaluated, 148 (60%) had normal mPAP, 36 (15%) had BoPAP**,** and 60 (25%) had definite PAH. Univariable logistic regression (ULR) showed the mean tricuspid regurgitation velocity in patients with BoPAP to be intermediate between normal mPAP and PAH. In the ULR analyses BoPAP versus normal mPAP and PAH versus BoPAP, the statistically significant predictors were, amongst others: demographic, clinical, pulmonary function, echocardiographic and hemodynamic variables.

**Conclusions:**

In this exploratory post-hoc analysis of the DETECT study population patients with BoPAP could be distinguished from patients with normal mPAP and PAH, and it appears that BoPAP may be an intermediate stage on the continuum between normal PA pressures and PAH.

**Electronic supplementary material:**

The online version of this article (doi:10.1186/s13075-014-0493-1) contains supplementary material, which is available to authorized users.

## Introduction

Systemic sclerosis (SSc, scleroderma) is an autoimmune connective tissue disorder characterized by inflammation, fibrosis and vasculopathy. Pulmonary arterial hypertension (PAH) is a leading cause of death in SSc [1,2]. Based on registry data, modern treatment has improved two-year survival estimates of SSc-associated PAH (SSc-PAH) from 40% [3] to 58% [4], a clear improvement which, however, lags gains seen in other forms of PAH [5-7]. Screening for SSc-PAH is capable of identifying earlier stages of the disease, resulting in earlier intervention that may improve survival [8]. Thus, early diagnosis and treatment of SSc-PAH is of paramount importance [9,10].

The DETECT study was a multi-center study that systematically evaluated 466 SSc patients at increased risk for development of SSc-PAH [11]. DETECT was the first SSc-PAH detection study to evaluate all subjects with right heart catheterization (RHC), the gold standard test for the diagnosis of PAH [9]. Detailed demographic, clinical, echocardiographic, serologic and functional testing data were also collected.

Measurements in healthy individuals show a normal mean pulmonary arterial pressure (mPAP) to be 14 ± 3.3 mmHg [12], with 2 SD extending the normal range up to 20.6 mm Hg. The consensus definition of PAH requires mPAP ≥25 mmHg and pulmonary artery wedge pressure (PAWP) of ≤15 mmHg [9,13]. Thus, a borderline range of mPAPs exists between 21 and 24 mm Hg that may represent an early, milder stage of pulmonary vasculopathy in those at high risk of developing PAH [13-15]. This concept is especially relevant in connective tissue diseases (CTD), such as SSc, where progressive vasculopathy is highly prevalent and may be an important distinction from idiopathic PAH [13]. The objective of this *post-*hoc analysis was to compare the demographics and clinical features of patients with normal PAP, borderline mPAP (BoPAP), and elevated PAP (PAH) in a large international cohort of patients with SSc who participated in the DETECT study. The identification of characteristics specific to SSc patients with BoPAP would facilitate future investigations into the natural history of this condition, and provide insights into the proportion of patients who develop PAH.

## Methods

### Study design

Our study was conducted in accordance with the Declaration of Helsinki and its amendments, followed the International Conference on Harmonization Guideline for Good Clinical Practice, and was approved by local institutional review boards/ethics committees (a complete list is included as Additional file 1). RHC and echocardiography protocols were standardized and conducted systematically, and serum laboratory testing as well as data management were performed centrally. Data quality was monitored rigorously. All patients provided written informed consent. Patients were eligible for inclusion in DETECT if they were aged ≥18 years and had: 1) a definite diagnosis of SSc [16] of >3 years’ duration from first non-Raynaud’s symptom; 2) a diffusing capacity of the lung for carbon monoxide (DLCO) <60% of predicted; 3) a forced vital capacity (FVC) ≥40% of predicted; and 4) not had pulmonary hypertension confirmed by RHC prior to enrolment.

### Current analysis population

Patients were included in the current analysis if they had: 1) a pulmonary artery wedge pressure (PAWP) ≤15 mmHg by RHC; 2) no significant interstitial lung disease (ILD; defined as FVC <60% or FVC between 60 and 70% with moderate-to-severe fibrosis on high resolution computed tomography); 3) no systemic hypertension (stage-I hypertension defined as systolic blood pressure ≥140 mmHg or diastolic blood pressure ≥90) [17]; and 4) no left atrial enlargement (defined as 3.9 cm for women and 4.1 cm for men) [18]. DETECT screened 646 SSc patients, and enrolled 488 SSc patients with 466 who underwent RHC. A total of 222 of 466 were excluded from the present analysis due to: 1) a pulmonary artery wedge pressure (PAWP) >15 mmHg or significant ILD (138 patients), and 2) an enlarged left atrium or stage-I or greater hypertension (84 patients). Eligible patients comprise the PAP analysis set and were categorized into three PAP groups for analysis, based on the mPAP by RHC: 1) normal mPAP (<21 mmHg); 2) BoPAP (mPAP 21 to 24 mmHg); and 3) PAH (mPAP ≥25 mmHg). Analysis of variables was performed using these three PAP groups.

### Data collection and analysis

The DETECT study collected 112 variables, collated into four groups: 1) demographic and clinical parameters (68 variables); 2) serum tests (13 variables); 3) electrocardiography (ECG) (3 variables); and 4) echocardiography (28 variables). In the present study, variables were selected for further analysis based on a review of numerical descriptive differences between the three groups, as well as input from the authors on feasibility of the variables and their clinical relevance to PAH. Two additional variables were computed: 1) the transpulmonary gradient (TPG) which is the difference between mPAP and left atrial pressure (estimated by the PAWP); and 2) the diastolic wedge gradient (DWG) which is the difference between diastolic PAP and left atrial pressure (estimated by the PAWP). The selected variables were described using summary statistics: sample size, mean, SD, median, upper and lower quartiles, minimum and maximum for quantitative data and frequencies (counts and percentages) for qualitative and categorical data. The distributions of the variables were compared using non-parametric tests: Wilcoxon rank-sum and chi-square/Fisher’s exact test for continuous and categorical data, respectively. Univariable logistic regression (ULR) analysis was performed using BoPAP versus normal mPAP and PAH versus BoPAP as binary outcomes in separate models. The odds ratio (OR) and 95% CI for each variable was calculated and statistical significance was examined by the Wald chi-square test. A Forest plot was constructed to display the odds ratios and the 95% CI for 13 variables in the BoPAP versus normal mPAP and PAH versus BoPAP groups. Variables were selected for the plot if they met the statistical significance criteria or had potential clinical utility as screening tests for BoPAP and PAH. RHC hemodynamics were used for exploratory purposes only, as they were part of the group definitions.

## Results

**Figure 1:**
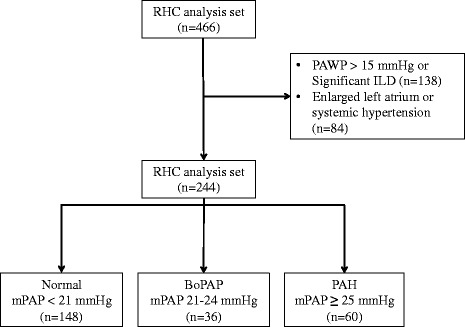
**Patient disposition.** This study focuses on the differences between the normal mean pulmonary arterial pressure (mPAP), borderline pulmonary arterial pressure (BoPAP) and pulmonary arterial hypertension (PAH) subgroups of the DETECT study. ILD, interstitial lung disease; PAWP, pulmonary artery wedge pressure; RHC, right heart catheterization.

Among the 244 SSc patients included in the PAP analysis set, 60% (n = 148) had a normal mPAP, 15% (n = 36) had BoPAP and 25% (n = 60) had an elevated mPAP (PAH) (Figure 1).

### Baseline characteristics of patients with normal mPAP, BoPAP and PAH

Statistically significant differences between the BoPAP and other groups were found in clinical, serologic, echocardiographic, and invasive hemodynamic parameters, including World Health Organization functional class (WHO FC) (PAH versus BoPAP), presence of peripheral edema (BoPAP versus normal mPAP), presence of telangiectasias (PAH versus BoPAP), ratio of FVC percent predicted/DLCO percent predicted (PAH versus BoPAP), TLC percent predicted (BoPAP versus normal mPAP), presence of anti-centromere antibodies (PAH versus BoPAP), log_10_ NT-proBNP (BoPAP versus normal mPAP), serum urate (PAH versus BoPAP), left atrium diameter (BoPAP versus normal mPAP, tricuspid annular plane systolic excursion (TAPSE, PAH versus BoPAP) and TR velocity (BoPAP versus both normal PAP and PAH; Table 1). Differences in the exploratory comparison of hemodynamic variables (PAWP, TPG, DWG and pulmonary vascular resistance (PVR)) were also noted.

**Table 1:** **Baseline characteristics of patients with normal mPAP, BoPAP and PAH**

	**Patient groups (N =244)**			***P*** **-value**	
**Variable**	**Normal mPAP (N =148)**	**BoPAP (N =36)**	**PAH (N =60)**	**BoPAP versus normal mPAP**	**PAH versus BoPAP**
**Demographic and clinical parameters**					
Age, years, N^a^	148	36	59		
Mean ± SD	54.3 ± 12.0	58.4 ± 9.5	61.6 ± 9.7		
Median, Q1 to Q3	55.0, 46.0 to 63.5	58.5, 52.5 to 63.5	62.0, 56.0 to 68.0		
Minimum, maximum	26.0, 78.0	43.0, 80.0	33.0, 80.0		
Gender, n	148	36	60		
Male, n (%)	18 (12.2)	6 (16.7)	12 (20.0)		
Female, n (%)	130 (87.8)	30 (83.3)	48 (80.0)		
WHO FC, N	139	34	60		<0.05
I/II, n (%)	127 (91.4)	28 (82.4)	37 (61.7)		
III/IV, n (%)	12 (8.6)	6 (17.6)	23 (38.3)		
6-minute walk distance, N	108	27	48		
Mean ± SD	422.4 ± 110.4	397.4 ± 102.3	391.4 ± 109.8		
Median, Q1 to Q3	432.5, 364.5 to 502.0	414.5, 341.0 to 472.0	395.0, 318.0 to 460.5		
SSc disease duration (years), N	147	36	60		
Mean ± SD	11.0 ± 8.0	10.0 ± 5.0	14.0 ± 12.0		
Median, Q1 to Q3	8.0, 5.0 to 13.0	9.0, 6.0 to 15.0	11.0, 6.0 to 16.0		
SSc subtype, N	146	36	59		
Limited, n (%)	90 (61.6)	20 (55.6)	44 (74.6)		
Diffuse, n (%)	45 (30.8)	13 (36.1)	10 (16.9)		
Overlap/mixed CTD, n (%)	11 (7.5)	3 (8.3)	5 (8.5)		
Presence of peripheral edema, n/N (%)	8/147 (5.4)	7/36 (19.4)	11/60 (18.3)	<0.01	
Presence of telangiectasias, n/N (%)	108/148 (73.0)	22/36 (61.1)	52/60 (86.7)		<0.005
FVC % predicted, N	148	36	60		
Mean ± SD	92.5 ± 17.6	88.7 ± 14.5	92.0 ± 19.7		
Median, Q1-Q3	91.9, 79.9 to 102.7	87.1, 80.0 to 96.5	89.5, 77.2 to 102.5		
FVC % predicted /DLCO % predicted, N	148	36	60		<0.01
Mean ± SD	1.9 ± 0.4	1.9 ± 0.4	2.3 ± 0.7		
Median, Q1 to Q3	1.8, 1.5 to 2.1	2.0, 1.5 to 2.2	2.2, 1.8 to 2.5		
TLC % predicted, N	127	28	54	<0.05	
Mean ± SD	92.8 ± 21.9	84.8 ± 14.4	88.1 ± 21.6		
Median, Q1 to Q3	92.5, 80.0 to 106.0	83.0, 74.0 to 95.0	87.3, 78.0 to 100.3		
**Serum laboratory tests**					
Presence of anti-centromere antibodies					<0.05
n/N (%)	49/141 (34.8)	10/34 (29.4)	30/56 (53.6)		
Log_10_ NT-proBNP (pg/ml), N	141	34	56	<0.05	
Mean ± SD	2.0 ± 0.5	2.2 ± 0.5	2.4 ± 0.5		
Median, Q1 to Q3	2.1, 1.7 to 2.3	2.2, 1.9 to 2.5	2.3, 2.0 to 2.8		
Serum urate, mg/100 ml, N	141	34	56		<0.01
Mean ± SD	4.7 ± 1.4	4.7 ± 1.3	5.6 ± 1.4		
Median, Q1 to Q3	4.4, 3.7 to 5.3	4.5, 3.7 to 5.4	5.6, 4.6 to 6.5		
Log_10_ estimated GFR	141	34	55		
	1.9 ± 0.1	1.9 ± 0.1	1.9 ± 0.1		
	1.9, 1.9 to 2.0	1.9, 1.8 to 2.0	1.9, 1.8 to 2.0		
**Echocardiography**					
Left atrium diameter, mm, N	148	36	60	<0.001	
Mean ± SD	30.2 ± 5.1	33.9 ± 3.6	31.8 ± 5.4		
Median, Q1 to Q3	31.0, 27.8 to 34.0	33.5, 31.0 to 37.0	32.0, 30.0 to 36.0		
Right atrium area, cm^2^, N	142	33	58		<0.05
Mean ± SD	12.8 ± 4.7	14.7 ± 5.9	16.8 ± 5.8		
Median, Q1 to Q3	12.0, 10.0 to 14.3	12.4, 10.5 to 18.0	15.8, 13.4 to 20.0		
TAPSE (mm), N	138	33	53		
Mean ± SD	23.2 ± 4.3	23.8 ± 5.1	21.1 ± 4.3		
Median, Q1 to Q3	23.7, 20.4 to 26.0	23.0, 20.0 to 27.0	20.8, 19.0 to 23.0		
TR velocity (m/s), N	140	35	58	<0.001	<0.05
Mean ± SD	2.3 ± 0.4	2.7 ± 0.3	3.0 ± 0.7		
Median, Q1 to Q3	2.3, 2.2 to 2.6	2.7, 2.5 to 2.9	2.9, 2.5 to 3.5		
**Electrocardiography**					
Presence of right axis deviation, n/N (%)	2/138 (1.4)	1/30 (3.3)	8/58 (13.8)		
**Right heart catheterization**					
PAWP, mmHg, N	148	36	60	<0.001	
Mean ± SD	7.7 ± 3.3	10.8 ± 3.0	9.9 ± 3.3		
Median, Q1 to Q3	8.0, 5.0 to 10.0	11.0, 9.0 to 13.0	10.5, 7.5 to 12.5		
TPG, mmHg, N	148	36	60	<0.001	<0.001
Mean ± SD	7.9 ± 2.7	11.6 ± 2.8	22.3 ± 9.7		
Median, Q1 to Q3	7.0, 6.0 to 10.0	11.0, 10.0 to 13.0	20.0, 15.0 to 28.0		
DWG, mmHg, N	148	36	60		<0.001
Mean ± SD	1.8 ± 2.7	3.0 ± 5.4	10.7 ± 8.1		
Median, Q1 to Q3	1.0, 0.0 to 3.0	2.0, 0.5 to 5.0	8.0, 5.0 to 17.0		
PVR, dyn.sec/cm^5^, N	148	36	60	<0.001	<0.001
Mean ± SD	128.0 ± 50.8	179.8 ± 58.7	375.8 ± 217.0		
Median, Q1 to Q3	121.5, 91.3 to 153.9	170.3, 147.8 to 187.8	295.5, 236.0 to 419.1		

^a^N for each variable represents the number of patients with available data. Statistical tests were Wilcoxon rank-sum for continuous data and chi-square/Fisher’s exact for categorical data. ^‘^These variables were analysed for completeness, but it should be borne in mind that they are part of the definition of the analysis groups. BoPAP, borderline pulmonary arterial pressure; CTD, connective tissue disease; DLCO, diffusing capacity of carbon monoxide; DWG, diastolic wedge gradient; FVC, forced vital capacity; GFR, glomerular filtration rate; NT-proBNP, N-terminal pro-brain natriuretic peptide; PAH, pulmonary arterial hypertension; PAWP, pulmonary artery wedge pressure; PVR, pulmonary vascular resistance; Q, quartile; SSc, systemic sclerosis; TAPSE, tricuspid annular plane systolic excursion; TLC, total lung capacity; TPG, transpulmonary gradient; TR, tricuspid regurgitation; WHO FC, World Health Organization functional class.

### Univariable logistic regression analysis

#### BoPAP versus normal PAP

**Figure 2:**
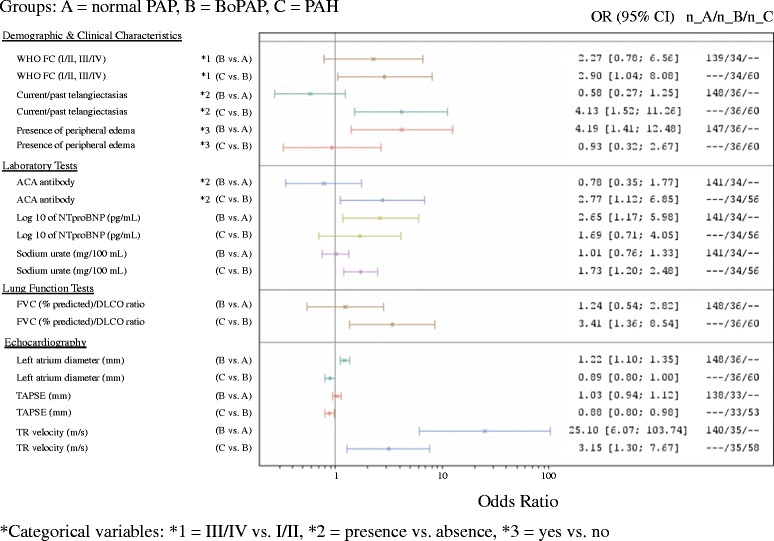
**Forest plot of odds ratios from univariable logistic regression analysis.** ACA antibody, anti-centromere antibody; BoPAP, borderline pulmonary arterial pressure; DLCO, diffusing capacity of carbon monoxide; FVC, forced vital capacity; NT-proBNP, N-terminal pro-brain natriuretic peptide; PAH, pulmonary arterial hypertension; PAP, pulmonary arterial pressure; WHO FC, World Health Organization functional class; TLC, total lung capacity; TR, tricuspid regurgitation.

As shown in Table 2 and Figure 2, ULR models of SSc patients with BoPAP versus normal mPAP identified the following variables as being statistically significant predictors of BoPAP (*P* <0.05): older age, presence of peripheral edema, a higher log_10_ NT-proBNP, greater left atrium diameter, and greater tricuspid regurgitation (TR) velocity. Of note, 6-minute walk distance was not statistically different.

**Table 2:** **Univariable logistic regression analysis of patients with BoPAP versus normal PAP and those with PAH versus BoPAP**

**Variable**	**Odds ratios (95% confidence interval)**		***P*** **-value**	
	**BoPAP versus normal mPAP**	**PAH versus BoPAP**	**BoPAP versus normal mPAP**	**PAH versus BoPAP**
**Demographic and clinical parameters**				
Age, years	1.03 (1.00, 1.07)	1.04 (0.99, 1.08)		
Gender, female versus male	0.69 (0.25, 1.89)	0.80 (0.27, 2.36)		
WHO FC, III/IV versus I/II	2.27 (0.78, 6.56)	2.90 (1.04, 8.08)		<0.05
6-minute walk distance	1.00 (0.99, 1.00)	1.00 (1.00, 1.00)		
SSc disease duration, years	0.99 (0.94, 1.04)	1.05 (1.00, 1.11)		
SSc disease subtype				<0.05
dcSSc versus lcSSc	1.30 (0.59, 2.85)	0.35 (0.13, 0.93)		
Overlap/mixed CTD versus lcSSc	1.23 (0.31, 4.81)	0.76 (0.17, 3.48)		
Presence of telangiectasias	0.58 (0.27, 1.25)	4.13 (1.52, 11.26)		<0.01
Presence of peripheral edema	4.19 (1.41, 12.46)	0.93 (0.32, 2.67)	<0.05	
FVC predicted	0.99 (0.97, 1.01)	1.01 (0.99, 1.04)		
FVC % predicted /DLCO % predicted	1.24 (0.54, 2.82)	3.41 (1.36, 8.54)		<0.01
TLC % predicted	0.98 (0.96, 1.00)	1.01 (0.99, 1.04)		
**Serum laboratory**				
Presence of anti-centromere antibodies	0.78 (0.35, 1.77)	2.77 (1.12, 6.85)		<0.05
Log_10_ NT-proBNP, pg/ml	2.65 (1.17, 5.98)	1.69 (0.71, 4.05)	<0.05	
Serum urate, mg/100 ml	1.01 (0.76, 1.33)	1.73 (1.20, 2.48)		<0.01
Log_10_ estimated GFR	0.54 (0.03, 9.44)	0.04 (0.00, 1.77)		
**Echocardiography**				
Left atrium diameter, mm	1.22 (1.10, 1.35)	0.89 (0.80, 1.00)	<0.001	<0.05
Right atrium area, cm^2^	1.07 (1.00, 1.14)	1.07 (0.99, 1.17)		
TAPSE, mm	1.03 (0.94, 1.12)	0.88 (0.80, 0.98)		<0.05
TR velocity, m/s	25.10 (6.07, 103.74)	3.15 (1.30, 7.67)	<0.0001	<0.05
**Electrocardiography**				
Right axis deviation	2.35 (0.21, 26.74)	4.64 (0.55, 38.99)		
**Right heart catheterization**				
PAWP, mmHg	1.39 (1.21, 1.61)	0.91 (0.79, 1.04)	<0.0001	
TPG, mmHg	1.57 (1.33, 1.85)	1.72 (1.35, 2.19)	<0.0001	<0.0001
DWG, mmHg	1.10 (0.99, 1.21)	1.27 (1.12, 1.44)		<0.0005
PVR, dyn.sec/cm^5^	1.01 (1.01, 1.02)	1.03 (1.01, 1.04)	<0.0001	<0.0001

BoPAP, borderline pulmonary arterial pressure; CTD, connective tissue disease; dcSSc, diffuse cutaneous SSc; DLCO, diffusing capacity of carbon monoxide; DWG, diastolic wedge gradient; FVC, forced vital capacity; GFR, glomerular filtration rate; lcSSc, limited cutaneous SSc; NT-proBNP, N-terminal pro-brain natriuretic peptide; PAH, pulmonary arterial hypertension; PAWP, pulmonary artery wedge pressure; PVR, pulmonary vascular resistance; SSc, systemic sclerosis; TAPSE, tricuspid annular plane systolic excursion; TLC, total lung capacity; TPG, transpulmonary gradient; TR, tricuspid regurgitation; WHO FC, World Health Organization functional class.

The RHC variables PAWP, TPG and PVR (which defined the groups) were all statistically significant, though DWG was not.

#### PAH versus BoPAP

ULR models of SSc patients with PAH versus BoPAP identified the following variables as being statistically significant (*P* <0.05) predictors of PAH: higher WHO FC (III or IV), diffuse versus limited disease subtype, the presence of telangiectasias, higher FVC percent predicted/DLCO percent predicted ratio, presence of anti-centromere antibodies, higher serum urate, a lower TAPSE and a higher TR velocity. As was the case with the BoPAP versus normal PAP analysis, 6-minute walk distance was not statistically different. As expected, the PAH group had a higher TPG, a higher DWG, and a higher PVR (Table 2 and Figure 2). Of note, the PAWP was not significantly different between these groups.

## Discussion

Expert consensus opinion from the recent 5th World Symposium on Pulmonary Hypertension acknowledges that a borderline mPAP between 21 and 24 mmHg in SSc patients is associated with ‘a high risk of future development of manifest PAHʼ, and recommends careful follow up of these patients [13]. In our current study of a large, well-defined, high-risk SSc cohort we identified clinical and laboratory features that differentiate the BoPAP group from groups with normal mPAP and PAH. Our study provides a crucial first step towards the development of future longitudinal studies of BoPAP, as we provide a panel of variables that can be tested to determine their prognostic utility.

Formalized early detection of SSc-PAH has been shown to identify milder forms of the disease, resulting in opportunities for earlier management [8,19]. As algorithms and practice guidelines are developed to improve early detection of SSc-PAH, it is likely that referrals for RHC will increase (in DETECT the rate increased from 40% to 62%). The increase in RHCs will likely result in more patients identified with both PAH and BoPAP. The finding of BoPAP is significant, as a number of recent studies have shown an association between BoPAP and worse clinical outcomes [14,15,20]. Thus, further characterization of BoPAP is important. In the current study we identify significant differences in clinical, serologic, echocardiographic and hemodynamic parameters useful in differentiating BoPAP from normal PAP and PAH from BoPAP groups.

A number of recent studies have shown an association between BoPAP and worse clinical outcomes. One small study compared SSc patients with mPAPs >17 but <25 mmHg (group mean of 20 ± 2 mmHg) to those with mPAPs ≤17 mmHg (group mean 14 ± 2 mmHg), and found the former group to be associated with both shorter mean 6-minute walk distances (396 ± 71 meters versus 488 ± 77 meters, *P* <0.005) and lower mean percent predicted peak oxygen consumption (VO_2_) values (76 ± 11% versus 90 ± 24%, *P* = 0.05) [20]. The PHAROS registry included 206 SSc patients at increased risk for PAH who underwent RHC [21]. After excluding patients with significant interstitial lung disease, a comparison of SSc patients with normal mPAP (group median 16 mmHg) and borderline mPAP (group median 23 mmHg) showed the latter group to have significantly higher right ventricular systolic pressures on echocardiogram, higher pulmonary vascular resistance and a higher transpulmonary gradient [14]. Follow-up data involving 24 PHAROS patients who underwent repeat RHC at a later date showed that 32% of patients with normal mPAP and 55% of patients with borderline mPAP developed resting PH (*P*-value not significant) 14.92 ± 9.23 and 12.19 ± 6.82 months after the first RHC, respectively [14]. Another analysis compared the outcomes of SSc patients with mPAP ≤20 mmHg to those with mPAPs of 21 to 24 mmHg [15]. Within 228 patients without ILD, 142 had normal mPAP and 86 had mPAP of 21 to 24 mmHg. Clinically indicated repeat RHCs were performed in 38 patients from each group and the hazard ratio for PAH diagnosis on the subsequent RHC for the borderline mPAP group compared to the normal mPAP group was 3.7 (95% CI 1.7, 8.0, *P* <0.001). Within the borderline mPAP group, 18.5% (95% CI 8.3, 28.7) developed PAH within 3 years, and 27.1% (95% CI 13.9, 40.3) developed PAH within 5 years [15].

In the current study we identified significant differences in clinical, serologic, echocardiographic and hemodynamic parameters useful in differentiating BoPAP from normal PAP and PAH from BoPAP groups. Across both comparisons, TR velocity was the variable most strongly associated with BoPAP (versus normal mPAP) and PAH (versus BoPAP).

Our analysis also identified additional variables capable of differentiating BoPAP from the PAH group: WHO functional class, SSc subtype, presence of telangiectasias, ratio of % predicted FVC/percent predicted DLCO, anti-centromere antibodies, and serum urate concentration. Some of these variables (telangiectasia [22], FVC/DLCO [22], anti-centromere antibodies [22-24], and serum urate [25]) have been useful in predicting SSc-PAH in other studies.

Invasive pressure measurements including TPG and DWG were explored, as these measurements have been addressed in other studies of BoPAP. Elevations in these gradients (≥12 mmHg and ≥5, respectively) [26] are believed consistent with pulmonary vascular disease, and thus, help separate individuals with PAH from those with pulmonary venous hypertension. As described previously [15], the TPG in our study was capable of separating the BoPAP group from the normal PAP and PAH groups. We also show the DWG to be useful in differentiating the BoPAP group from the PAH group, and it has the added benefit of being able to differentiate PAH from post-capillary PH [26]. Though not in the abnormal range, the mean TPG and DWG measurements for the BoPAP group were intermediate between the normal PAP and PAH groups, perhaps suggesting that a subset of SSc patients with BoPAP may have early pulmonary vasculopathy.

In the present study we excluded patients with overt pulmonary venous hypertension based upon the finding of a PAWP >15 mmHg by RHC. It is important to acknowledge that occult pulmonary venous hypertension (OPVH) due to left heart disease may have been present in a proportion of patients who underwent a RHC while in a state of volume depletion, and were thus found to have underestimated left-heart filling pressures [27]. One study involving patients with scleroderma found that 6 out of 24 patients who originally met the criteria for PAH by RHC were subsequently diagnosed with OPVH after a fluid challenge [28]. As a fluid challenge was not included in our RHC study protocol, we minimized the likelihood of including patients with OPVH by excluding individuals with conditions commonly associated with OPVH: stage I or greater systemic hypertension and/or abnormally enlarged left atrium. Future BoPAP studies should consider the use of evocative maneuvers such as fluid challenge or exercise in order to identify patients with OPVH.

Our study has significant strengths. Ours is the first BoPAP study to mandate a diagnostic RHC in all subjects, allowing us to assign individuals into groups (normal mPAP, BoPAP and PAH) based on rigorous, objective criteria. Standardization of the study protocol and centralization of serum laboratory testing and data management assured homogeneity. In addition, it is the largest, most complete cohort of patients evaluated for SSc-PAH, providing us a sufficient sample size to conduct our analyses.

Our study has important limitations. Results are cross-sectional, so longitudinal follow up to determine the incidence of PAH in the BoPAP group over time was not determined. Our inclusion criteria included a DLCO <60% to enrich for a higher likelihood of PAH. Thus, the groups analyzed in our study represent a high-risk SSc group, and may not be representative of the general SSc population. Future studies are needed in order to expand our findings into the general SSc population. Our study was not designed to compare and model relationships involving the BoPAP sub-group; thus, multivariable logistic regression for this post-hoc sub-analysis could be misleading, and was not performed. In addition, the three sub-groups analyzed (normal mPAP, BoPAP and PAH) were defined using mPAP (a continuous variable). Various pairwise sub-groups using binary logistic regression may provide a different set of predictive covariables for each multiple regression model that may be inaccurate.

## Conclusion

In conclusion, our study identifies clinical, echocardiographic, hemodynamic, pulmonary function, and serologic variables that allow SSc patients with BoPAP to be differentiated from SSc patients with normal mPAPs and PAH. Future longitudinal studies designed to reassess patients with BoPAP at pre-designated time points would be the ideal means of validating the prognostic value of our findings. Such studies would allow further characterization of the natural history of BoPAP, including the rate and frequency of conversion to pre-capillary or post-capillary PH. Our exploratory analyses of hemodynamic data suggest that the TPG and DWG should continue to be evaluated as potential predictors for the development of PAH in the SSc population.

## Additional file

Additional file 1:
**Institutional ethics committees and review boards.**

## Abbreviations

BoPAP: borderline elevation of the mean pulmonary arterial pressure
CTD: connective tissue disease
DLCO: diffusing capacity of the lung for carbon monoxide
DWG: diastolic wedge gradient
FVC: forced vital capacity
GFR: glomerular filtration rate
HFpEF: heart failure with preserved ejection fraction
ILD: interstitial lung disease
mmHg: millimeters of mercury
mPAP: mean pulmonary arterial pressure
NT-proBNP: N-terminal pro-brain natriuretic hormone
OPVH: occult pulmonary venous hypertension
OR: odds ratio
PAH: pulmonary arterial hypertension
PAWP: pulmonary artery wedge pressure
PVR: pulmonary vascular resistance
RHC: right heart catheterization
ROC AUC: receiver operating characteristic area under the curve
SSc: systemic sclerosis (scleroderma)
SSc-PAH: scleroderma-associated pulmonary arterial hypertension
TAPSE: tricuspid annular plane systolic excursion
TLC: total lung capacity
TPG: transpulmonary gradient
TR: tricuspid regurgitation
ULR: univariable logistic regression
VO_2_: oxygen consumption
WHO FC: World Health Organization functional class

## References

[1] Steen VD, Medsger TA (2007). Changes in causes of death in systemic sclerosis, 1972-2002. Ann Rheum Dis.

[2] Tyndall AJ, Bannert B, Vonk M, Airo P, Cozzi F, Carreira PE, Bancel DF, Allanore Y, Muller-Ladner U, Distler O, Iannone F, Pellerito R, Pileckyte M, Miniati I, Ananieva L, Gurman AB, Damjanov N, Mueller A, Valentini G, Riemekasten G, Tikly M, Hummers L, Henriques MJ, Caramaschi P, Scheja A, Rozman B, Ton E, Kumánovics G, Coleiro B, Feierl E (2010). Causes and risk factors for death in systemic sclerosis: a study from the EULAR Scleroderma Trials and Research (EUSTAR) database. Ann Rheum Dis.

[3] Stupi AM, Steen VD, Owens GR, Barnes EL, Rodnan GP, Medsger TA (1986). Pulmonary hypertension in the CREST syndrome variant of systemic sclerosis. Arthritis Rheum.

[4] Condliffe R, Kiely DG, Peacock AJ, Corris PA, Gibbs JS, Vrapi F, Das C, Elliot CA, Johnson M, DeSoyza J, Torpy C, Goldsmith K, Hodgkins D, Hughes RJ, Pepke-Zaba J, Coghlan JG (2009). Connective tissue disease-associated pulmonary arterial hypertension in the modern treatment era. Am J Respir Crit Care Med.

[5] Kuhn KP, Byrne DW, Arbogast PG, Doyle TP, Loyd JE, Robbins IM (2003). Outcome in 91 consecutive patients with pulmonary arterial hypertension receiving epoprostenol. Am J Respir Crit Care Med.

[6] Clements PJ, Tan M, McLaughlin VV, Oudiz RJ, Tapson VF, Channick RN, Rubin LJ, Langer A (2012). The pulmonary arterial hypertension quality enhancement research initiative: comparison of patients with idiopathic PAH to patients with systemic sclerosis-associated PAH. Ann Rheum Dis.

[7] Rubenfire M, Huffman M, Krishnan S, Seibold JR, Schiopu E, McLaughlin VV (2013). Survival in systemic sclerosis with pulmonary arterial hypertension has not improved in the modern era. Chest.

[8] Humbert M, Yaici A, de Groote P, Montani D, Sitbon O, Launay D, Gressin V, Guillevin L, Clerson P, Simonneau G, Hachulla E (2011). Screening for pulmonary arterial hypertension in patients with systemic sclerosis: clinical characteristics at diagnosis and long-term survival. Arthritis Rheum.

[9] McLaughlin VV, Archer SL, Badesch DB, Barst RJ, Farber HW, Lindner JR, Mathier MA, McGoon MD, Park MH, Rosenson RS, Rubin LJ, Tapson VF, Varga J (2009). ACCF/AHA 2009 expert consensus document on pulmonary hypertension a report of the American College of Cardiology Foundation Task Force on Expert Consensus Documents and the American Heart Association developed in collaboration with the American College of Chest Physicians; American Thoracic Society, Inc.; and the Pulmonary Hypertension Association. J Am Coll Cardiol.

[10] Khanna D, Gladue H, Channick R, Chung L, Distler O, Furst DE, Hachulla E, Humbert M, Langleben D, Mathai SC, Saggar R, Visovatti S, Altorok N, Townsend W, FitzGerald J, McLaughlin VV (2013). Recommendations for screening and detection of connective tissue disease-associated pulmonary arterial hypertension. Arthritis Rheum.

[11] Coghlan JG, Denton CP, Grunig E, Bonderman D, Distler O, Khanna D, Muller-Ladner U, Pope JE, Vonk MC, Doelberg M, Chadha-Boreham H, Heinzl H, Rosenberg DM, McLaughlin VV, Seibold JR (2013). Evidence-based detection of pulmonary arterial hypertension in systemic sclerosis: the DETECT study. Ann Rheum Dis.

[12] Kovacs G, Berghold A, Scheidl S, Olschewski H (2009). Pulmonary arterial pressure during rest and exercise in healthy subjects: a systematic review. Eur Respir J.

[13] Hoeper MM, Bogaard HJ, Condliffe R, Frantz R, Khanna D, Kurzyna M, Langleben D, Manes A, Satoh T, Torres F, Wilkins MR, Badesch DB (2013). Definitions and diagnosis of pulmonary hypertension. J Am Coll Cardiol.

[14] Bae S, Saggar R, Bolster MB, Chung L, Csuka ME, Derk C, Domsic R, Fischer A, Frech T, Goldberg A, Hinchcliff M, Hsu V, Hummers L, Schiopu E, Mayes MD, McLaughlin V, Molitor J, Naz N, Furst DE, Maranian P, Steen V, Khanna D (2012). Baseline characteristics and follow-up in patients with normal haemodynamics versus borderline mean pulmonary arterial pressure in systemic sclerosis: results from the PHAROS registry. Ann Rheum Dis.

[15] Valerio CJ, Schreiber BE, Handler CE, Denton CP, Coghlan JG (2013). Borderline mean pulmonary artery pressure in patients with systemic sclerosis: transpulmonary gradient predicts risk of developing pulmonary hypertension. Arthritis Rheum.

[16] Masi AT, Subcommittee for Scleroderma Criteria of the American Rheumatism Association Diagnostic and Therapeutic Criteria Committee (1980). Preliminary criteria for the classification of systemic sclerosis (scleroderma). Subcommittee for scleroderma criteria of the American Rheumatism Association Diagnostic and Therapeutic Criteria Committee. Arthritis Rheum.

[17] Chobanian AV, Bakris GL, Black HR, Cushman WC, Green LA, Izzo JL, Jones DW, Materson BJ, Oparil S, Wright JT, Roccella EJ (2003). The Seventh Report of the Joint National Committee on Prevention, Detection, Evaluation, and Treatment of High Blood Pressure: the JNC 7 report. JAMA.

[18] Lang RM, Bierig M, Devereux RB, Flachskampf FA, Foster E, Pellikka PA, Picard MH, Roman MJ, Seward J, Shanewise JS, Solomon SD, Spencer KT, Sutton MS, Stewart WJ (2005). Recommendations for chamber quantification: a report from the American Society of Echocardiography’s Guidelines and Standards Committee and the Chamber Quantification Writing Group, developed in conjunction with the European Association of Echocardiography, a branch of the European Society of Cardiology. J Am Soc Echocardiogr.

[19] Galie N, Rubin L, Hoeper M, Jansa P, Al-Hiti H, Meyer G, Chiossi E, Kusic-Pajic A, Simonneau G (2008). Treatment of patients with mildly symptomatic pulmonary arterial hypertension with bosentan (EARLY study): a double-blind, randomised controlled trial. Lancet.

[20] Kovacs G, Maier R, Aberer E, Brodmann M, Scheidl S, Troster N, Hesse C, Salmhofer W, Graninger W, Gruenig E, Rubin LJ, Olschewski H (2009). Borderline pulmonary arterial pressure is associated with decreased exercise capacity in scleroderma. Am J Respir Crit Care Med.

[21] Hinchcliff M, Fischer A, Schiopu E, Steen VD (2011). Pulmonary Hypertension Assessment and Recognition of Outcomes in Scleroderma (PHAROS): baseline characteristics and description of study population. J Rheumatol.

[22] Johnson SR, Fransen J, Khanna D, Baron M, van den Hoogen F, Medsger TA, Peschken CA, Carreira PE, Riemekasten G, Tyndall A, Matucci-Cerinic M, Pope JE (2012). Validation of potential classification criteria for systemic sclerosis. Arthritis Care Res (Hoboken).

[23] Thenappan T, Shah SJ, Rich S, Gomberg-Maitland M (2007). A USA-based registry for pulmonary arterial hypertension: 1982-2006. Eur Respir J.

[24] Gladue H, Steen V, Allanore Y, Saggar R, Saggar R, Maranian P, Berrocal VJ, Avouac J, Meune C, Trivedi M, Khanna D (2013). Combination of echocardiographic and pulmonary function test measures improves sensitivity for diagnosis of systemic sclerosis-associated pulmonary arterial hypertension: analysis of 2 cohorts. J Rheumatol.

[25] Njaman W, Iesaki T, Iwama Y, Takasaki Y, Daida H (2007). Serum uric Acid as a prognostic predictor in pulmonary arterial hypertension with connective tissue disease. Int Heart J.

[26] Naeije R, Vachiery JL, Yerly P, Vanderpool R (2013). The transpulmonary pressure gradient for the diagnosis of pulmonary vascular disease. Eur Respir J.

[27] Robbins IM, Hemnes AR, Pugh ME, Brittain EL, Zhao DX, Piana RN, Fong PP, Newman JH (2014). High prevalence of occult pulmonary venous hypertension revealed by fluid challenge in pulmonary hypertension. Circ Heart Fail.

[28] Fox BD, Shimony A, Langleben D, Hirsch A, Rudski L, Schlesinger R, Eisenberg MJ, Joyal D, Hudson M, Boutet K, Serban A, Masetto A, Baron M (2013). High prevalence of occult left heart disease in scleroderma-pulmonary hypertension. Eur Respir J.

