# Supplementary material for: Borderline pulmonary arterial pressure in systemic sclerosis patients: a post-hoc analysis of the DETECT study
Source: Arthritis Res Ther. 2014 Dec 10;16(6):493. doi: 10.1186/s13075-014-0493-1 (PMC4299685; doi:10.1186/s13075-014-0493-1)
Supplement: Additional file 1: — Institutional ethics committees and review boards. [file 13075_2014_493_MOESM1_ESM.docx]

Institutional Ethics Committees and Review Boards

**Austria**

Ethik-Kommission Der Medizinischen Universität Wien Und Des Allgemeinen Krankenhauses Der Stadt Wien Akh

Ethikkommission der Medizinischen Universität Graz, LKH-Universitätsklinikum

A.ö. Krankenhaus der Elisabethinen Linz

**Bosnia and Herzegovina**

Klinički centar Univerziteta u Sarajevu Etički komitet

**Canada**

Office of Research Ethics, University of Western Ontario

Mount Sinai Hospital Research Ethics Board

Comité d'Éthique de la Recherche du CHUM

Hôpital Général Juif Research Ethics Committee

Capital Health Research Ethics Board

Ethics Office University of Saskatchewan

UBC-Providence Health Care Research Institute Office of Research Services

Faculty of Medicine, University of Calgary Office of Medical Bioethics

University of Manitoba Bannatyne Campus Research Ethics Boards

Health Research Ethics Board Administration Office, University of Alberta

**China**

Ethics Committee of the Chinese Academy of Medical Sciences Beijing Union Medical College Hospital

**Czech Republic**

Etická komise pro multicentrické klinické hodnocení Fakultní nemocnice v Motole

Etická komise RNDr

**Germany**

Ethikkommission der medizinischen Fakultät Heidelberg

Landesärztekammer Hessen Ethikkommission

Ethikkommission der Medizinischen Fakultät der Technischen Universität Dresden

Ethik-Kommission des Fachbereichs Medizin der Johann Wolfgang Goethe-Universität

Ethik-Kommission der Medizinischen Fakultät der Friedrich-Alexander-Universität Erlangen-Nürnberg

Ethikkommission der Ärztekammer Westfalen-Lippe und der med. Fakultät der WWU Münster

Ethik-Kommission an der Medizinischen Fakultät der Universität Leipzig

Ethik-Kommission der Medizinischen Fakultät der Universität zu Köln

Ethik-Kommission der Albert-Ludwigs-Universität Freiburg

"Ethikkommission Charité-Universitätsmedizin Berlin

Ethikkommission an der Medizinischen Fakultät der Rheinischen Friedrich-Wilhelms-Universität Bonn

Ethikkommission an der Medizinischen Fakultät Ernst-Moritz-Arndt-Universität Greifswald

Landesärztekammer Rheinland-Pfalz Ethikkommission

**Hungary**

Eg.szs.gügyi Tudományos Tanács

Regional Research Ethics Committee, University of Debrecen

Regional Research Ethics Committee, Institute of Medical Microbiology and Immunity Science

**The Netherlands**

Commissie Mensgebonden Onderzoek

**Norway**

REK VEST

**Poland**

Komisja Bioetyczna przy OIL w Białymstoku

**Romania**

Colegiul Medicilor din Romania Bioethical Committee

Ethical Committee, University of Medicine and Pharmacy

**Russia**

Ethics Committee at Federal Service on Surveillance in Healthcare and Social Development of Russian Federation

Scientific Research Rheumatology Institute Ethics Committee

**Slovakia**

Eticka komisia

**Spain**

Comité Ético Investigación Clínica

Comité Ético de Investigación Clínica, Servicio de Farmacología Clínica

**Switzerland**

Kantonal Ethik-Kommission

Comité départemental d'éthique de medicine interne-médecine communautaire

**Turkey**

T.C.Sağlık Bakanlığı İlaç Dışı Klinik Araştırmalar Etik Danışma Kurulu

İstanbul Üniversitesi Tıp Fakültesi

**United Kingdom**

Royal Free Hospital and Medical School, Research Ethics Committee Chairman

**United States**

BioMedical Research Alliance of New York, LLC (BRANY) Institutional Review Board

Boston University Medical Center Office of the Institutional Review Board

Georgetown University Institutional Review Board

Institutional Review Board, Human Research Review Committee, Medical College of Wisconsin

IRB & Compliance Office, Los Angeles Biomedical Research Institute at Harbor UCLA Medical Center

Johns Hopkins Medicine School of Medicine Office of Human Subjects Research Institutional Review Board

Mayo Clinic Institutional Review Board

Medical University of South Carolina Office of Research Integrity

Spectrum Health Research and Human Rights Committee/IRB

UCLA Medical Institutional Review Board Office for Protection of Research Subjects

The University of Toledo Department of Human Research Protection Biomedical Institutional Review Board

Thomas Jefferson University Office of Human Research Division of Human Subjects Protection Institutional Review Board

University of Connecticut Health Center Human Subjects Protection Office Institutional Review Board

University of Illinois at Chicago Office for the Protection of Research Subjects (OPRS)

University of Michigan Medical School Institutional Review Board

University of Minnesota Research Subjects' Protection Programs

Western Institutional Renewal Board, Olympia, WA
